# Psychometric Properties of Spanish Version of the Three-Factor Eating Questionnaire-R18 (Tfeq-Sp) and Its Relationship with Some Eating- and Body Image-Related Variables

**DOI:** 10.3390/nu6125619

**Published:** 2014-12-04

**Authors:** Ignacio Jáuregui-Lobera, Patricia García-Cruz, Rocío Carbonero-Carreño, Alejandro Magallares, Inmaculada Ruiz-Prieto

**Affiliations:** 1 Department of Molecular Biology and Biochemistry Engineering (Nutrition and Bromatology), Pablo de Olavide University, Carretera de Utrera s/n, Seville 41013, Spain; 2 Behavioral Sciences Institute, Fernando IV, 24-26, Seville 41011, Spain; E-Mails: patricia-gcr@hotmail.com (P.G.-C.); inma.irp@gmail.com (I.R.-P.); 3 IES Atenea, Mairena del Aljarafe, Seville 41927, Spain; E-Mail: rociocarbonero@gmail.com; 4 Social Psychology Department, School of Psychology, Spanish Open University (UNED), Madrid 28040 Spain; E-Mail: amagallares@psi.uned.es

**Keywords:** eating behavior, TEFQ, psychometric validation, dietary restraint, obesity, eating disorders

## Abstract

The aims of this study were to analyze the psychometric properties of the Spanish version of the Three-Factor Eating Questionnaire-R18 (TFEQ-SP), as well as determine its validity by evaluating the relationship of the TFEQ-SP with different parameters related to body mass index, weight perception, perception of physical fitness, self-esteem, and food intake, as well as with weight control-related variables. A total of 281 participants (aged 18.38 ± 6.31) were studied. The factor analysis yielded three factors: cognitive restraint (CR), uncontrolled eating (UE), and emotional eating (EE). The internal consistency of the TFEQ-SP was determined by means of Cronbach’s α coefficient, with values ranging between 0.75 and 0.87. Higher scores on CR were found in women (*p* < 0.5), overweight/obese participants (*p* < 0.001), participants with lower self-esteem (*p* < 0.05), participants who overestimated their weight (*p* < 0.001), participants who weighed themselves frequently (*p* < 0.001) and those who were about to go on a diet (*p* < 0.001). Higher EE scores were found in participants with lower self-esteem scores (*p* < 0.05), among participants with a poorer perception of their physical fitness (*p* < 0.01) and when participants were about to diet (*p* < 0.05). Higher scores on UE were observed in case of poorer perception of physical fitness (*p* < 0.05). The validation study of the TFEQ-SP meets the requirements for measuring the three different facets of eating behavior: CR, UE, and EE.

## 1. Introduction

Interactions between eating behavior and health are a field of study absolutely necessary to improve prevention programs, as well as treatments, regarding eating disorders and obesity among other disturbances [1]. In the study of eating behaviors, the concept of dietary restraint is relevant, highlighting the regulation of food intake in order to control weight and body shape. That control based on restrictions may cause consequent overeating episodes and eating disorders, and overweight and obesity at long-term [2]. Along with dietary restraint, other eating behaviors have been described such as loss of control over intake and overeating as consequence of emotional distress [3].

Many instruments (e.g., the Restraint Scale, the Three-factor Eating Questionnaire, the Dutch Eating Behaviour Questionnaire) have been designed to measure these different eating behaviors, especially dietary restraint, trying to find the most appropriate one [4,5,6]. Apart from the design of instruments, it is necessary to study the validity of them across different populations (obese and normal weight persons, clinical and non-clinical samples, groups of different sex and age, *etc.*) [2].

A special topic refers to the relation between eating behaviors and diet/body weight, which to some extent remains unclear [7]. It is usually thought that weight gain begins to occur when the intake is higher than the energy expenditure. Nevertheless, there are contradictory data with respect to this topic. Thus a negative association between intake of sugars and body weight has been reported, the heritability of body mass index is well known (up to 80% in Western populations) and there is evidence that genetic factors contribute to the variation in the responses to fatty acids [8,9,10,11]. In addition, different eating behavior styles might also be determined by genetic effects [12]. Thus, it is not clear whether different eating behaviors cause weight gain or, *vice versa*.

First of all it is relevant to measure these different eating behaviors. In this regard, the Stunkard and Messick Three-Factor Eating Questionnaire (TFEQ) and the Three-Factor Eating Questionnaire Revised 18-item version (TFEQ-R18) are the most used and studied tools [2,5]. On the basis that lengthy questionnaires can be shortened and at the same time maintain their validity by means of a specific selection of items, Karlsson *et al.* [2] designed the TFEQ-R18. This instrument is comprised of three subscales corresponding to (a) cognitive restraint (CR), defined as conscious restriction of food intake aimed to control body weight and/or to promote weight loss; (b) emotional eating (EE), which refers to the tendency to eat more than usual due to a loss of control over intake with a subjective feeling of hunger; and (c) uncontrolled eating (UE), defined as the inability to resist emotional cues, eating as a response to different negative emotions. These subscales are comprised of six, three, and nine items, respectively. The validity of this instrument has been studied by considering different variables, such as measurements of well-being, anthropometric parameters or physical activity-related variables (e.g., Karlsson *et al.* [2]).

The general aim of the present study was to analyze the psychometric properties, factor structure and internal consistency of the Three-Factor Eating Questionnaire Spanish version (TFEQ-SP). A further objective was to analyze the validity of the EFEQ-SP by studying the relationships between the TFEQ-SP and different variables, such as body weight, weight perception, body shape, perception of the physical fitness, self-esteem and different behaviors to control weight. In addition possible gender differences were analyzed.

## 2. Methods

### 2.1. Participants

Participants were a group of students, which comprised a total of 281, none of whom had a history of psychological disorders. This group included 119 women (42.3%) and 162 men (57.7%), with a mean age of 18.38 ± 6.31 years (their ages ranging from 12–27). Their mean Body Mass Index (BMI) was 22.98 ± 4.66 (range 14.6–37.7). No inclusion/exclusion criteria were applied with respect to age range and BMI range for this study. Those students who were receiving psychological support for adaptive/academic reasons were excluded. The students were recruited from Instituto de Enseñanza Secundaria Atenea (Mairena del Aljarafe, Seville, Spain).

### 2.2. Measures

#### 2.2.1. Three-Factor Eating Questionnaire-R18

This questionnaire is a shortened version of the original 51-item TFEQ [5]. The Spanish version of the TFEQ-R18 (TFEQ-SP) was obtained by conducting a translation and back translation procedure, without any overlap across the members who performed the translation and the back translation. The questionnaire measures three different aspects of eating behavior: (a) restrained eating (defined as conscious restriction of food intake aimed to control body weight and/or to promote weight loss); (b) uncontrolled eating (the tendency to eat more than usual due to a loss of control over intake with a subjective feeling of hunger); and (c) emotional eating (inability to resist emotional cues, eating as a response to different negative emotions). The questionnaire comprises of 18 items that are measured on a 4-point response scale (definitely true: 1, mostly true: 2, mostly false: 3, definitely false: 4) and items scores are summated into subscale scores: CR, UE and EE. Previous studies have reported that TFEQ-R18 has adequate internal consistency reliability coefficients for the three subscales, as well as for the whole questionnaire (ranging between 0.75 and 0.87) [2].

#### 2.2.2. Weight- and Body Shape-Related Factors

Anthropometric measurements. BMI was calculated as the relationship between weight (in kg) and height squared (in m). Weight and height were taken in individual sessions, with the participants in the standing position, barefoot, and in light garments. A stadiometer “Añó-Sayol Atlántida S13” model was used. Body composition was analyzed by means of a tetrapolar bio-impedance analyzer (Tanita TBF 300, Tanita Europe, Sindelfingen, Germany) with millimeter accuracy (0.1 kg for weight, 0.1% for body fat mass) and capacity up to 200 kg (0–200 kg) and 1%–75% of body fat mass.

Weight perception. In order to measure the weight perception, participants responded to this question: What do you think of yourself in terms of weight? Possible responses were: very overweight, slightly overweight, about the right weight, slightly underweight and very underweight.

Self-weighing frequency participants were asked about their self-weighing frequency: several times per day, daily, several times per week, weekly, occasionally.

Body shape. Participants were asked to choose, based on the standard figural stimuli developed by Stunkard and Stellar (1990) [13]: Which silhouette is closest to your usual appearance?

#### 2.2.3. Diet-Related Factors

Dieting. Participants were asked whether or not they were dieting at the moment (yes/no), the reason or reasons for going on that diet (aesthetic reasons, healthy reasons—others than losing weight—or only with the specific objective of losing weight) and the intention of keeping on dieting or being about to do it (yes/no).

Food intake. Food intake was analyzed by means of a 24-h dietary recall covering three days: two weekdays and one weekend day (to capture the usual variability of the diet). It has been reported that three 24-h recalls produce the best estimate of energy intake [14]. In order to minimize the loss of information (memory effect) the weekdays were the two weekdays before the assessment and the weekend day was the previous Sunday. A quantitative analysis (energy intake, % of fats, lipid profile, % of carbohydrates, % of proteins and the daily amount of fiber) was performed by means of the “Dial” software [15].

#### 2.2.4. Physical Activity-Related Factors

International Physical Activity Questionnaire (IPAQ). The short version was used in this study, which provides information on the time spent on walking, moderate-intensity activities, vigorous-intensity activities and sedentary activities. Regarding the psychometric properties, the short IPAQ has shown about 0.65 reliability and adequate validity, having a reasonable agreement with the long form (*r* = 0.67, 95% CI: 0.64 to 0.70) [16].

Self-Reported Physical Fitness. Participants were asked about their physical fitness perception (How do you consider your current physical fitness looks like?) and they were classified as perceiving themselves as possessing a poor, fair, average, good or excellent physical fitness.

#### 2.2.5. Self-Esteem Scale (SES)

The scale comprises of 10 items that are scored using a Likert format (from strongly agree to strongly disagree): the higher the score, the higher the degree of self-esteem. The Spanish version of the instrument shows adequate internal consistency (Cronbach’s alpha coefficient = 0.87), test-retest reliability (*r* = 0.72) and construct validity, and it was used in the current study [17,18].

### 2.3. Procedure

Before conducting the study, the ethics approval was obtained from the Behavioral Sciences Institute (Res-BSI 09/13, September 2013). Thereafter, the project was transferred to the Director of the IES Atenea, who, after accepting it, submitted that project to the students’ council, the academic staff and the students’ fathers and mothers association obtaining the approval by all of them. Once this approval was achieved, a timetable was established to collect the data during a week (3–7 March 2014), thus, avoiding evaluation periods, in which the possible distress could have influenced the study.

In order not to alter the routines of the classes in the center, the questionnaires were filled in during the periods assigned to tutorials and the anthropometric measures were collected in hours devoted to physical education. All students were invited to participate, and participation was completely voluntary. For the participants under 18 the parents’ informed consent was compulsory.

Measurements were taken individually and participants were instructed how to complete the questionnaires. In addition, a professional (nutritionist or psychologist) was in charge to resolve any doubts about the tasks when carrying them out. None of the participants received any kind of reward for their participation and anonymity was guaranteed.

### 2.4. Statistical Analyses

Conventional descriptive statistics (M, SD) were used to describe continuous variables and percentages for the categorical ones (*N*, %). The Kolmogorov-Smirnov test was used to determine whether the data fitted a normal distribution and the Levene test was used to explore the similarity between variances. Taking into account the results of these tests, comparisons were analyzed using an analysis of variance (ANOVA). Parametric Pearson correlation coefficients (*r*) were calculated in order to analyze the association between some variables considered in this study. In case of categorical variables, the χ^2^-test was applied. All analyses were performed using SPSS^®^ version 20.0.0 (IBM Corporation, Armonk, NY, USA).

## 3. Results

Descriptive data with respect to the main categorical and continuous variables used in this study are shown in Table 1 and Table 2.

**Table 1:** Descriptives statistics. Categorical variables (*n* = 281).

Variables	*N* (%)
*Sex*	
Males	162 (57.7%)
Females	119 (42.3%)
*Weight perception*	
Very overweight	24 (8.5%)
Slightly overweight	75 (26.7%)
About the right weight	148 (52.7%)
Slightly underweight	31 (11%)
Very underweight	3 (1.1%)
*Body shape (silhouettes)*	
BMI 17	17 (6%)
BMI 19	80 (28.5%)
BMI 21	71 (25.3%)
BMI 23	68 (24.2%)
BMI 25	31 (11%)
BMI 27	14 (5%)
*Dieting*	
Yes	37 (13.2%)
No	244 (86.8%)
*Planning to go on a diet for aesthetic reasons*	
Yes	123 (43.8%)
No	151 (53.7%)
?	7 (2.5%)
*Weigh themselves*	
Several times per day	3 (1.1%)
Daily	9 (3.2%)
Several times per week	13 (4.6%)
Weekly	26 (9.3%)
Occasionally	230 (81.8%)

**Table 2:** Descriptives statistics. Continuous variables (*n* = 281). Mean ± SD.

Variables	Mean ± SD
Age	18.38 ± 6.31
*BMI*	22.98 ± 4.66
Males	23.82 ± 4.77
Females	21.83 ± 4.26
*Body fat percentage*	22.14 ± 12.85
Males	18.77 ± 14.56
Females	26.65 ± 8.24
Physical activity (IPAQ: MET/min/day)	536.63 ± 465.61
Self-esteem score (SES)	31.62 ± 5.83
Calorie intake	1752.02 ± 483.84
*TFEQ-SP*	
Cognitive restraint	11.69 ± 4.11
Uncontrolled eating	17.50 ± 6.20
Emotional eating	5.14 ± 2.65

### 3.1. Construct Validity (Factor Structure)

A factor analysis was performed using the principal components analysis with varimax rotation. Several indicators of the high degree of inter-relationship between the variables confirmed the relevance of this analysis. Bartlett’s test of sphericity gave χ^2^ = 1582.55 (*p* < 0.0001), while the Kaiser-Meyer-Olkin (KMO) index of sample adequacy was 0.840. The number of factors was determined by considering those with eigenvalues above 1, through examination of the scree plot, and with the parallel analysis [19,20,21,22]. Items with a factor loading ≥0.45 and which loaded on a single factor were maintained. With respect to the parallel analysis, three of the eigenvalues in the factor analysis were greater than the average eigenvalues in the parallel analysis. As result, the best solution for the analysis of the 18 items of the TFEQ-SP yielded three factors that corresponded to the scales previously identified: UE (nine items), EE (three items) and CR (six items). These three factors accounted for 53.12% of the variance.

Table 3 shows the rotated factor loadings, the explained variance and the accumulated variance.

The first factor, which explains 19.14% of the total variance, comprises nine items that refer to the uncontrolled eating scale. The second factor explains 17.68% of the total variance and consists of a further three items that refer to the emotional eating part of the scale. Finally, the third factor explains 16.30% of the total variance and refers to the cognitive restraint part of the scale.

**Table 3:** Factor structure (principal components with varimax rotation) and explained variance of the TFEQ-SP.

Item	Item content	Uncontrolled Eating	Cognitive Restraint	Emotional Eating
14	How often do you feel hungry?	0.68		
13	I am always hungry enough to eat at any time	0.67		
1	When I smell a delicious food, I find it very difficult to avoid eating even if I have just finished a meal	0.63		
9	I am always hungry so it is hard for me to stop eating before I finish the food on my plate	0.63		
7	When I see a real delicacy, I often get so hungry that I have to eat right away	0.62		
4	Sometimes when I start eating, it seems I am unable to stop	0.55		
5	Being with someone who is eating often makes me hungry enough to eat also	0.55		
8	I get so hungry that my stomach often seems like a bottomless pit	0.51		
17	Do you go on eating binges in spite of you not being hungry?	0.47		
11	I consciously hold back at meals in order not to gain weight		0.75	
12	I do not eat some foods because they make me grow fat		0.73	
16	How likely are you to consciously eat less than you want to?		0.71	
2	I deliberately take small helpings as means of controlling my weight		0.71	
15	How frequently do you avoid stocking up on tempting foods?		0.65	
18	On a scale of 1–8, where 1 means no restraint in eating and 8 means total restraint, what number would you give yourself?		0.58	
6	When I feel unwell (depressive, unhappiness), I often overeat			0.83
10	When I feel lonely, I relieve myself by eating			0.83
3	When I feel anxious, I find myself eating			0.50
Explained variance		19.14	17.68	16.30
Accumulated variance		19.14	36.82	53.12

Loadings < 0.45 are left blank.

### 3.2. Internal Consistency

The internal consistency of the TFEQ-SP and its three scales was analysed by means of Cronbach’s alpha coefficient, which is commonly used as an estimate of the reliability. The UE scale had α = 0.87 and the corresponding values for the EE scale and CR scale were α = 0.79 and α = 0.74. Overall, the questionnaire (TFEQ-SP) yielded α = 0.83. With respect to the item-scale convergent validity and item-discriminant validity, correlations between items assigned to a scale and the other two scales were <0.30 except for one item of the UE, which correlated 0.53 to one item in the EE scale. Thus, 17 out of 18 items met the criteria for both convergent and discriminant validity. The correlation intra-scales ranged between 0.46 and 0.85.

### 3.3. Appealing Features

Regarding to the appealing features the TFEQ-SP was easily administered and scored, and it required only a few minutes to be completed (M = 3.42 min) with a range between 1.27 and 6.12 min.

### 3.4. Criterion Validity

#### 3.4.1. TFEQ-SP and Weight- and Body-Related Factors

Considering normal weight (59.4%), overweight/obese participants (27.1%) and underweight participants (13.5%) there were some significant differences with respect to the CR scale (underweight = 10.22 ± 4.6; normal weight = 11.43 ± 4.07; overweight/obese = 12.68 ± 3.37; *F*_3, 277_ = 5.78; *p* < 0.001). *Post hoc* tests revealed higher scores on CR in the group of overweight/obese participants with respect to the rest. No significant differences were found between normal weight and underweight participants. Taking into account the participants’ body weight perception, there were some significant differences on the CR scale (*F*_4, 276_ = 9.20; *p* < 0.001). Those participants who perceived their body weight as normal or slightly underweight showed lower scores on CR (10.79 and 9.97 respectively) than those who perceived their weigh slightly or very overweight (13.53 and 13.54 respectively). Considering body weight and body weight perception there were not any significant differences on the UE and EE scores. Significant correlations were found between CR and body fat mass (*r* = 0.27; *p* < 0.001) and between CR and BMI (*r* = 0.21; *p* <0.01).

Participants who had chosen silhouettes corresponding to BMI between 17 and 21 showed lower scores on CR scale than those who had chosen figural stimuli associated to BMI between 23 and 27 (9.29–11.19 *vs.* 13.51–14.54, respectively) (*F*_5, 275_ = 8.55; *p* < 0.001). Again there were not any differences with regards to UE and EE. The percentage of students identified with the silhouettes associated to BMI from 23 to 27 was 67.57.

Among the students, a total of 18.15% weighed themselves frequently (weekly or more), the girls being much more notoriously prone to adopt this pattern than boys (55% *vs.* 45%). Among these girls, 28.51% were dieting at the moment of the study while 39.13% of the boys who weighed themselves were dieting too. Broadly those students who weighed themselves frequently (weekly or more) showed a lower level of self-esteem (29.41 *vs.* 32.15; *F*_1, 279_ = 9.68; *p* < 0.01). Participants who controlled their weight daily or weekly showed higher scores on CR than the rest of students (11.11 and 13.58 *vs.* 11.24; *F*_4, 276_ = 4.89; *p* < 0.01). No differences were found with regards to UE and EE.

#### 3.4.2. TFEQ-SP and Diet-Related Factors

Among those who went on a diet (13.2%) at the moment of the study, the score on CR was higher than in the rest of participants (14.92 *vs.* 11.19 respectively), this differences being statistically significant (*F*_1, 279_ = 28.14; *p* < 0.001). No differences were found with respect to UE and EE. Participants who were about to go on a diet showed higher scores on CR than those who were not (12.50 *vs.* 10.65; *F*_1, 279_ = 11.43; *p* < 0.01). In the group of participants who were about to go on a diet, 70.88% had taken their decision based on aesthetic reasons. Among them, the scores on CR and EE were significantly higher (13.34 *vs.* 10.20; *F*_1, 77_ = 21.80; *p* < 0.001).

The correlations between eating behavior and food intake were not significant except in the case of daily calorie intake and cholesterol intake. The correlation between CR and daily calorie intake was significant (*r* = −0.20; *p* < 0.05), as well as the correlation between CR and cholesterol intake (*r* = −0.16; *p* < 0.05). For the percentages of carbohydrates, proteins and fats (except cholesterol) as well as the daily amount of fiber no differences were found.

#### 3.4.3. TFEQ-SP and Physical Activity-Related Factors

The different levels of physical activity were not significantly correlated with the TFEQ-SP scales, except in the case of sedentary activities, which was positive and significantly correlated to UE (*r* = 0.22; *p* < 0.01). Considering the self-reported physical fitness, those students who considered to possess a poor-fair physical fitness showed higher scores on CR (12.69 *vs.* 10.99; *p* < 0.05). Comparing participant who perceived their physical fitness as poor with the rest of students, the latter showed lower scores on UE (21.44 *vs.* 17.26; *p* < 0.05). The same occurred with respect to EE, with higher scores among those who had a poor self-reported physical fitness (7.11 *vs.* 4.91; *p* < 0.05).

#### 3.4.4. TFEQ-SP and Self-Esteem

Participants with higher self-esteem (above the median) had lower scores on CR than those with a lower self-esteem (*F*_1, 279_ = 6.10; *p* < 0.05), as well as lower scores on EE (*F*_1, 279_ = 5.92; *p* < 0.05). No differences were found with regards to UE. In fact, significant correlations were found between self-esteem and CR (*r* = 0.21; *p* < 0.01), as well as between self-esteem and EE (*r* = 0.19; *p* < 0.0.5).

### 3.5. Gender Differences, Age

Generally speaking women showed higher scores on CR than men (12.37 *vs.* 11.18; *F*_1, 279_ = 5.68; *p* < 0.05). A positive and significant correlation was found between age and EE (*r* = 0.16; *p* < 0.05).

## 4. Discussion

As in previous studies on TFEQ [1,2] the present research on TFEQ-SP obtained three factors that correspond to the following scales: cognitive restraint (CR), emotional eating (EE) and uncontrolled eating (UE) with six, three, and nine items, respectively. With regards to our factor analysis, in the proposed factor structure, items 4 and 17 had similar loadings on both UE and EE. There may be a certain degree of confusion between some “uncontrolled” and “emotional” aspects of eating behavior at least with respect to the items which refer to the difficulty to stop after starting eating and to go on eating binges despite not feeling hungry. Based on the previous study of Karlsson *et al.* [2], there is a good theoretical ground for assigning these two items to the UE scale.

In general, the validation study of the Spanish version of the TFEQ meets the requirements for measuring the three different facets of eating behavior: the tendency to eat more than usual due to a loss of control over intake with a subjective feeling of hunger (uncontrolled eating), the conscious restriction of food intake aimed to control body weight and/or to promote weight loss (cognitive restraint, restrained eating) and inability to resist emotional cues (emotional eating). The analysis of reliability shows that the Spanish version has adequate internal consistency, both as regards the total questionnaire and for each one of the scales and factors.

The factor structure of TEFQ has proved to be valid in both obese samples and non-clinical samples [2,23] and our study adds new data in this regard. With respect to the cognitive restraint scale, its construct validity is well established [5,23,24] and the current study confirms this validity. Something different from a conceptual point of view seems to occur considering the uncontrolled and emotional scales. As was mentioned, two items showed loadings, which seemed to be controversial. It has been noted that the fact of the emotional scale being comprised of three items could be a problem. In addition the idea of loss of control over eating is correlated with perceived hunger while restraint eating is negatively associated to both loss of control and hunger [2]. Moreover, does loss of control require a previous state of restriction? If so, hunger, loss of control and restraint could have some inter-relationships, which might influence the results when measuring eating behaviors. Nevertheless, it has been noted that loss of control (disinhibition) is an important eating behavior trait that does not require a previous state of restriction [25]. The concept of emotional eating has been supported by the relations between negative affects and failures in the attempts to reduce weight [26]. The link between negative affects and eating behavior might be based on at least three different variables. First of all, personality traits could be involved in that link. Other possibility is that some psychopathological variables could be the link between negative affects and eating. Finally, eating as consequence of emotional problems might be mediated by a previous dietary restraint. With respect to the TFEQ, the fact that the emotional scale consists of three items leads to the idea of increasing that number of items in order to assess emotional eating involving new items, which reflect a more diverse picture of negative affects.

With respect to the criterion validity, this study based on TFEQ includes different variables, such as anthropometrical, psychological, related to weight and body shape perception, related to physical activity and self-reported physical fitness and those related to diet and food intake. Participants with higher weight and those who perceived themselves as overweight showed higher scores on CR. The connection between higher BMI and higher scores on CR has been reported previously [27,28]. In this regard it has been noted that cognitive dietary restraint is not consistently linked to body weight-adiposity, whereas rigid and flexible restraint are oppositely related to obesity [29]. In our study women showed higher scores on CR than men as in the study of Provencher *et al.* [29], this suggesting that gender might mediate associations between eating behaviors and anthropometric parameters. With respect to body image, it has been reported that obese adolescents used cognitive restraint more than normal-weight adolescents as a strategy for regulating their diet and were less satisfied with their body image [30]. To some extent our study confirms this results. Those participants identified with silhouettes corresponding to the highest BMI showed the highest scores on CR. With respect to weight expectations, it has been reported that women with a more realistic happy BMI showed a higher score for flexible restraint [31]. That could be reasonably related to the fact of dieting as stated by Megalaki *et al.* [30]. Those students who went on a diet at the moment of our study (13.2%) had higher scores on CR than the rest of participants. Other authors have reported that current and past dieters had higher scores for cognitive dietary restraint and disinhibition compared with non-dieters [32]. Among those identified with silhouettes corresponding to BMI between 23 and 27, 67.57% went on a diet. The fact of being about to dieting gave similar results and the same applied when the reason of dieting was aesthetic. Thus, body weight, body image and dieting seem to be clearly related to CR as a style of eating behavior.

De Lauzon *et al.* [1] analyzed the relationship between the different eating behaviors as measured by the TFEQ and food intake (by means of a food frequency questionnaire). As a result they found out some differences: CR scores were positively associated to healthy food groups and negatively associated to some other foods (French fries, sugar and confectionery); UE was positively associated to energy-dense foods (in men) and fruit; and EE was positively associated to snacks. Our study did not consider the food groups but food intake (macronutrients and fiber) by means of a 24-h dietary recall. Our results only support a negative association between CR and daily calorie intake and total cholesterol. For the rest of fats, as well as proteins, carbohydrates and fiber we did not find significant differences with regards to eating behaviors. Considering the concept of disinhibition intake, an internal factor (eating in response to internal cues) seems to be associated with poorer long-term weight loss outcomes [33]. In the case of exercise-induced weight management, eating behaviors, such as disinhibition and restraint, seem to have an important role in the relationship between exercise and energy balance [34].

In our sample, a total of 18.15% weighed themselves frequently (weekly or more), the girls being more inclined to weigh themselves than boys (55% *vs.* 45%). This result is similar to the reported by Quick *et al.* [35]. These authors found that girls who weighed themselves frequently were more likely to diet, get engaged in unhealthy and extreme weight control behaviors, use unhealthy muscle-enhancing behaviors, and have lower self-esteem and greater body dissatisfaction. Our study does not find a similar result but the contrary with more men dieting among those who were usual self-weighers. From a psychological point of view, Quick *et al.* [28] reported a lower self-esteem in the case of girls who weighed themselves quite often. A similar result was found in our research for the complete sample (men and women). Again CR appears linked to other variables of this study, such as weighing and self-esteem. Participants with lower self-esteem and who were frequent self-weighers showed higher scores on CR. Other authors have reported that TFEQ factors are related to psychological well-being [31].

The concept of “extreme weight conditions” has been introduced recently with regards to cognitive functions. Fagundo *et al.* [36] have hypothesized that cognitive deficits observed in anorexia nervosa and obese patients might partially be an expression of their incapacity to successfully regulate reward and punishment, which might affect the planning of every day function. Their cognitive performance and their eating behavior seem to have similarities. These two extreme weight conditions could make people eat differently, thus, the weight condition could predict the eating behavior and not *vice versa*. With respect to obesity this has been hypothesized by other authors [1,27] at least referring to CR. Our results show that students with a higher BMI refer more CR but this is not the case for those with lower BMI. In our research we can refer to low weight condition (low BMI) but not to specific eating disorders (not explored for the proposal of this research) so we cannot support the idea about similarities between anorexia nervosa and obesity with respect to eating behaviors (CR).

Bearing in mind that CR has been found to be associated with body/weight-related variables, dieting and food intake and psychological variables such as self-esteem, it should be appropriate to introduce here the concept of pathogenic *vs.* non-pathogenic weight control behaviors. On the one hand pathogenic weight-control behavior is used to “manage emotions/feelings, weight and body size” and includes unhealthy ways of controlling weight, gradual or rapidly. On the other hand non-pathogenic weight control is purely functional and targeted (for example with sport specific objectives) [37,38,39]. Gradual weight-control behavior is a more careful, continuous way, for example through restraint, selective eating or exercising over a longer period of time while rapid weight-control is characterized by a fast change of body weight through behaviors within a short time period (e.g., purging behaviors). In both gradual and rapid ways the increase of eating disorders is a fact. Thus, despite not being the proposal of this study, our results on the different variables linked to CR eating behavior should lead to future research based on the specific risk for the development of eating disorders/obesity. The idea of exercising as a way to control weight shows another interesting result in our study. Students who perceive themselves as having a poor-fair physical fitness also show higher CR and those who consider their physical fitness as poor show higher scores on UE and EE. In addition UC is correlated with sedentary activities. Thus, the perception of physical fitness seems to be linked to different eating behaviors and might be associated to different weight control behaviors.

In conclusion, CR is associated to overweight/obesity and with the perception of being overweight as well as with the silhouettes corresponding to the highest BMI. In addition students with the highest scores on CR usually diet more frequently than the rest do. The diet of those who have a high score on CR is a bit different, with lower calorie intake and less amount of cholesterol. The weight control behavior is also more frequent among students with high CR scores and self-reported physical fitness is poorer in this group of students. On the contrary, persons with higher self-esteem show lower scores on CR. Above all women seem to have higher scores on this eating behavior style. This CR style is a robust construct across different samples as was reported by Karlssson *et al.* [2], as well as in the current study. In our research UE only was different among the participants with poorer self-reported physical fitness and it was correlated to sedentary activities. With regards to EE, the scores were higher in those students who were about to go on a diet, something that seems to connect with the idea of pathogenic and gradual weight control behavior. Finally there was a positive correlation between EE and age, which possibly suggests an increasing link between emotions/problems and several coping strategies (in this case a specific way of eating could be considered as a “strategy” to cope with negative emotions) [40,41].

This study has some limitations. With respect to the food intake, the data generated by using a 24-h dietary recall may not represent the long-term dietary habits of the participants. Thus, finding an association between food intake and other variables should be taken into account carefully. Another point to be considered is the age of the participants. Our study focused on a sample of participants aged 18 ± 6.31 while previous research is mainly based on participants in their 40s–50s [29,31,32,36]. In this regard comparisons should be considered with caution. In this study body dissatisfaction was not calculated from the body shape figural measure and no other variables were considered specifically focused on body dissatisfaction. Future works could be carried out considering the relationship between body dissatisfaction and eating behaviors as assessed by the TFEQ-SP. Bearing in mind previous research, future studies based on the TFEQ-SP should explore the links between eating behaviors and other variables in specific populations as it has been made previously in other studies [31,33,34,36].

## 5. Conclusions

The construct validity of the TFEQ-SP is adequate in these participants of Spanish population with a wide-ranging body weight so the instrument seems to be a useful tool to measure different eating behavior styles. Connections of the three factors of the questionnaire (especially cognitive restraint) with different variables such as anthropometrical, psychological, related to weight and body shape perception, related to physical activity and self-reported physical fitness and those related to diet and food intake, were, in the most of the cases, in accordance with previous research. TFEQ-SP has adequate psychometric properties and it is a valid measure of cognitive restraint, uncontrolled eating and emotional eating in the Spanish population of the age range studied in here.

## Appendix

### Spanish Version of the Three Factor Eating Questionnaire-R18 (TFEQ-SP)

Adaptation and validation by Jáuregui-Lobera, I.; García-Cruz, P.; Carbonero-Carreño, R.; Magallares, A. and Ruiz-Prieto, I.; 2014.

(1) Cuando huelo una comida deliciosa me resulta muy difícil no probarla, incluso si acabo de terminar mi comida.
(2) Deliberadamente tomo pequeñas cantidades de comida como medio para controlar mi peso.
(3) Cuando me siento ansioso/a (nervioso/a) sin darme cuenta me encuentro comiendo.
(4) A veces cuando empiezo a comer parece que no puedo parar.
(5) Estar con alguien mientras come me hace sentir hambre como para ponerme a comer también.
(6) Cuando me siento mal (depresivo, infeliz) suelo comer demasiado.
(7) Cuando veo algo muy exquisito me entra tanta hambre que tengo que comerlo en ese mismo momento.
(8) Me siento tan hambriento/a que mi estómago a menudo parece un pozo sin fondo.
(9) Siempre tengo hambre, de modo que para mí es difícil parar de comer hasta que acabo la comida del plato.
(10) Cuando me siento solo/a me consuelo comiendo.
(11) Me controlo conscientemente en las comidas para no ganar peso.
(12) No suelo comer algunos alimentos porque me hacen engordar.
(13) Siempre siento tanta hambre como para poder comer en cualquier momento.
(14) ¿Con qué frecuencia te sientes hambriento/a?
(15) ¿Con qué frecuencia evitas almacenar alimentos muy tentadores/apetecibles?
(16) ¿Con qué probabilidad comes conscientemente menos de lo que quieres?
(17) ¿Continúas comiendo excesivamente aunque no tengas hambre?
(18) En una escala de 1 a 8, donde 1 significa no restringir la ingesta y 8 significa restricción total, ¿con qué número te valorarías a ti mismo/a?
